# Rhamnolipid-augmented medium enhances growth of *Dehalobium chlorocoercia* on chloroethenes and polychlorinated biphenyls

**DOI:** 10.1128/aem.00841-26

**Published:** 2026-08-03

**Authors:** Vijay Hemmadi, Randhir S. Makkar, Jace W. Jones, Christopher W. Marshall, Allen R. Place, Harold D. May, Kevin R. Sowers

**Affiliations:** 1 Department of Marine Biotechnology, Institute of Marine and Environmental Technology, University of Maryland Baltimore County 14701, Baltimore, Maryland, USA; 2 Enzylignol Inc, Ladson, South Carolina, USA; 3 Department of Pharmaceutical Sciences, University of Maryland School of Pharmacy, Baltimore, Maryland, USA; 4 Department of Biological Sciences, Marquette University 5505, Milwaukee, Wisconsin, USA; 5 Institute of Marine and Environmental Technology, University of Maryland Center for Environmental Sciences, Baltimore, Maryland, USA; 6 Hollings Marine Laboratory, Medical University of South Carolina 2345, Charleston, South Carolina, USA; Michigan State University, East Lansing, Michigan, USA

**Keywords:** biosurfactants, glycolipids, *Pseudodesulfovibrio *sp., perchloroethene, bioremediation

## Abstract

Polychlorinated biphenyls (PCBs) and chlorinated ethenes, such as perchloroethene (PCE), are persistent environmental contaminants that pose significant ecological and health risks. *Dehalobium chlorocoercia* strain DF-1 can reductively dechlorinate both PCEs and PCBs, offering a sustainable remediation strategy. However, its large-scale cultivation has been limited by dependence on an undefined growth-stimulatory factor produced by *Pseudodesulfovibrio* sp. (DSV). Filtrates of DSV-autoclaved cultures passed through a 10-kDa cutoff filter enhanced growth-linked dechlorination of DF-1, indicating that the active component is a small-molecular-weight, nonsecretory, thermostable compound. Acid precipitation of the autoclaved DSV extract yielded a glycolipid-enriched fraction that also supported DF-1 growth; in parallel, the model biosurfactant surfactin stimulated DF-1 activity, consistent with a biosurfactant-mediated effect. Liquid chromatography coupled to high-resolution tandem mass spectrometry analysis of the acid-precipitated fraction detected ions and fragmentation patterns consistent with multiple rhamnolipid congeners, including mono- and di-rhamnolipids with C8–C12 acyl chains. Both glycolipid-enriched extract and commercial rhamnolipids substituted for DSV extracts, stimulating DF-1 growth-linked reductive dechlorination of PCE, 2,3,4,5-tetrachlorobiphenyl (PCB 61), and 2,2′,3,3′,4,5,5′,6-Octachlorobiphenyl (PCB 199) in a concentration-dependent manner. Maximal DF-1 growth-linked dechlorination rates were observed at submicellar rhamnolipid concentrations, whereas higher concentrations were inhibitory. Rhamnolipid supplementation reduced growth lag and increased DF-1 biomass and dechlorination rates by solubilizing hydrophobic electron acceptors, thereby increasing their bioavailability in the medium. These findings suggest that rhamnolipid-type biosurfactants in *Pseudodesulfovibrio* sp. extracts have a role in enhancing the growth of DF-1 and demonstrate that commercial rhamnolipid supplementation can replace undefined coculture extracts, enabling reproducible large-scale cultivation of DF-1 for bioaugmentation of PCB- and PCE-contaminated environments.

**IMPORTANCE:**

Polychlorinated biphenyls (PCBs) and chlorinated ethenes remain widespread contaminants that are difficult to remove with conventional technologies, creating a need for scalable bioremediation strategies. *Dehalobium chlorocoercia* DF-1 is one of the few isolates available that can reductively dechlorinate both PCBs and PCE, but its application has been hindered by reliance on undefined extracts from a *Pseudodesulfovibrio* sp. This study shows that the key stimulatory activity is associated with rhamnolipid-type glycolipid biosurfactants released from *Pseudodesulfovibrio* sp. cells upon lysis. Glycolipid-enriched extracts and defined commercial rhamnolipids enhance DF-1 growth-linked dechlorination of PCE and PCB congeners in a dose-dependent manner, primarily by increasing the bioavailability of hydrophobic electron acceptors. By replacing complex coculture extracts with a defined biosurfactant supplement, these findings remove a major barrier to reproducible, large-scale cultivation of DF-1 and advance the feasibility of bioaugmentation for PCB- and PCE-contaminated environments.

## INTRODUCTION

Polychlorinated biphenyls (PCBs) and chlorinated ethenes, such as perchloroethene (PCE), are among the most hazardous and persistent organic pollutants (POPs). Their stability is derived from strong carbon-chlorine bonds, high hydrophobicity, and chemical inertness-characteristics that not only make these compounds ideal for a wide range of industrial applications but also underlie their environmental recalcitrance and bioaccumulation in terrestrial and aquatic organisms (1–3). Chronic exposure is associated with neurological, endocrine, reproductive, and immune dysfunction, leading to the ban of PCB production in 1976 and their designation as priority pollutants under the Stockholm Convention (4–6). Similarly, PCE and trichloroethene (TCE), once widely used as degreasers and dry-cleaning solvents, are now prevalent groundwater contaminants at Superfund National Priorities List (NPL) sites due to improper disposal and their recognized carcinogenicity (7, 8).

Conventional remediation methods are costly, labor-intensive, and ecologically disruptive, underscoring the need for sustainable alternatives. Microbial bioremediation offers a promising strategy, leveraging organohalide-respiring bacteria (OHRB) that reductively dechlorinate more extensively chlorinated pollutants under anaerobic conditions (9–11). This process detoxifies compounds and generates less-chlorinated congeners with reduced bioaccumulation potential and greater susceptibility to aerobic degradation (12). PCB dechlorination has been reported worldwide, with isolates such as *Dehalococcoides* and *Dehalobium* as key organisms (9, 13–15).

*Dehalobium chlorocoercia* strain DF-1 (DF-1) is notable for dechlorinating doubly flanked meta- and para-chlorines of PCBs and the chloroethenes PCE and trichloroethene (6, 11, 16). Phylogenetically, *Dehalobium* belongs to the class Dehalococcoidia (phylum Chloroflexota) and is grouped among the obligate organohalide-respiring bacteria that conserve energy exclusively via reductive dehalogenation of organohalides as terminal electron acceptors. DF-1 is a strictly anoxic, organohalide-respiring bacterium; growth has only been observed under anaerobic conditions with halogenated ethenes or PCBs as electron acceptors and formate or H₂/CO₂ as electron donors, and no fermentative metabolism has been reported. In contrast to facultative OHRB, such as *Geobacter*, *Desulfitobacterium*, or *Sulfurospirillum*, DF-1 has not been shown to use alternative non-halogenated electron acceptors or to grow in the absence of organohalides (11, 17–19). Unlike other reported PCB-dechlorinating species that require poorly soluble hydrogen gas for growth (0.16 mg/L), DF-1 grows on highly soluble sodium formate (97.2 g/L), yielding higher biomass at large volumes required for field bioaugmentation. The ability of DF-1 grown with PCE to retain the ability to grow and dechlorinate PCBs provides additional advantages: (i) DF-1 grows more rapidly and attains higher culture densities than with PCB, (ii) PCE is highly volatile and can be removed from the culture by sparging with nitrogen before harvesting, and (iii) cultures grown with PCE can be directly transferred to PCBs with a negligible lag in growth (11, 16, 18, 20–23). Bioaugmentation trials have demonstrated their potential for *in situ* remediation of PCB-contaminated sediments (6, 20–22, 24). Payne et al. (22, 25) addressed this challenge in the field by developing a “synergistic system” in which activated carbon pellets inoculated with DF-1 and the aerobic PCB degrader *Burkholderia xenovorans* LB400 promote sequential anaerobic-aerobic degradation of PCBs. Activated carbon concentrates PCBs near a DF-1 biofilm, which removes chlorine substituents that otherwise shield the biphenyl ring and prevent aerobic biphenyl-mineralizing bacteria from accessing the substrate. By eliminating this steric barrier, DF-1 makes PCBs amenable to aerobic mineralization by biphenyl-degrading microorganisms, enabling complete degradation (16, 20–22, 24–29). Although this system has demonstrated feasibility in pilot-scale applications, its broader implementation is constrained by DF-1’s inherently slow growth, limited biomass yields, and dependence on an undefined extract from its co-culture partner *Pseudodesulfovibrio* sp. (DSV). The undefined nature of this stimulatory factor limits the reproducibility and scale-up of biomass, posing a significant bottleneck for field applications (11).

Initial observations suggested that the active factor(s) in the *Pseudodesulfovibrio* sp. extract exhibit small size, thermostability, and non-proteinaceous properties, features consistent with biosurfactants (11). Limited bioavailability of hydrophobic pollutants is a well-known limitation in bioremediation, and biosurfactants can alleviate this by enhancing solubility, reducing interfacial tension, and promoting bioavailability (30–32). Previous studies have linked lipopeptides and glycolipids to enhanced PCB degradation, although mainly in sediment or mixed cultures (33–36). Notably, sulfate-reducing bacteria such as *Desulfovibrio desulfuricans,* often reported in OHRB enrichments, have been shown to synthesize biosurfactants under anaerobic conditions, supporting the hypothesis that DF-1 growth stimulant is a biosurfactant (37–39).

Rhamnolipids are glycolipid biosurfactants, best-characterized in *Pseudomonas aeruginosa* and also produced by some sulfate-reducing bacteria, and are composed of one or two rhamnose sugars linked to β-hydroxy fatty acid chains. Their low critical micelle concentration, amphiphilic structure, and thermal stability make them effective at enhancing the bioavailability of hydrophobic pollutants at low concentrations, thereby supporting higher microbial biomass and apparent degradation rates (35, 36, 40, 41).

This study aims to characterize the growth-stimulatory factor in the *Pseudodesulfovibrio* sp. extract that enhances DF-1 growth-linked dechlorination. Based on preliminary evidence that the factor is small, thermostable, and non-proteinaceous, it was hypothesized to be a biosurfactant. To test this hypothesis, both acid-precipitated *Pseudodesulfovibrio* sp. extract, enriched in glycolipid biosurfactants, and commercial rhamnolipids were evaluated to determine whether rhamnolipid-type biosurfactants account for, and can substitute for, the undefined DSV extract in supporting DF-1 growth and activity. Establishing a defined replacement would eliminate reliance on complex coculture components and enable reproducible large-scale cultivation of DF-1 in defined media, thereby advancing the scale-up of cells for anaerobic bioremediation of PCB- and PCE-contaminated environments.

## MATERIALS AND METHODS

### Growth and maintenance of cultures

*D. chlorocoercia* (DF-1) was anaerobically cultured in estuarine medium without sulfate (E-Cl medium), as previously described (16), with the following modifications: sodium formate (30 mM) was supplied as the electron donor. Either 2,3,4,5-tetrachlorobiphenyl (PCB 61; purity >99%, Accustandard) or perchloroethene (PCE; purity >99%, Fluka) was dissolved in acetone, and 0.1% (vol/vol) was added to the medium for final concentrations of 50 mg/L (sol. 16 µg/L) and 17 mg/L (sol. 150 mg/L), respectively (42, 43). The undissolved PCB fraction acts as a reservoir, and because of the low solubility, the dissolved bioavailable fraction does not reach toxic levels (43, 44). PCE was added to cultures at a concentration below the solubility threshold that was optimal for the growth of DF-1 (45). *Pseudodesulfovibrio* sp. extract (1% vol/vol) was provided as a growth stimulant, while titanium (III) nitrilotriacetate (TiNTA; 0.5 mM) and L-cysteine·HCl·H₂O (1 mM) were added as reductants.

Cultures for inoculum preparation were maintained in 160 mL serum bottles containing 50 mL of E-Cl medium. The bottles were sealed with 20 mm Teflon-coated butyl stoppers (West Pharmaceutical, Inc.) and secured with aluminum crimp seals. The headspace contained a mixture of N_2_:CO_2_ (4:1). Cultures were incubated statically at 29°C in the dark. For PCE-grown cultures, periodic purging was performed at 15- to 20-day intervals using a N_2_:CO_2_ mixture, and PCE was replenished as needed.

*Pseudodesulfovibrio* sp. isolated from the original DF-1 coculture by Wu, Watts, et al. (19) was anaerobically cultured in an estuarine medium with the following additions: sodium lactate (10 mM) and sodium sulfate (10 mM) as an electron donor and acceptor, respectively, and cysteine (3 mM) as the only reductant. Cultures were maintained in 160 mL serum bottles and incubated as described above for DF-1 (24).

### Monitoring PCE and PCB dechlorination activity of DF-1

Reductive dechlorination activity of chloroethenes was monitored by sampling 100 μL of the headspace with a gastight glass syringe (SGE, Inc.) every 2–4 days. Chloroethene congeners, including PCE, trichloroethene (TCE), and dichloroethene (DCE), were quantified using an HP6890 gas chromatograph equipped with a flame ionization detector (GC-FID; Agilent Technologies) and an HP-5MS capillary column (30 m × 0.320 mm × 0.25 µm; Agilent Technologies). Calibration curves for quantification were prepared using a six-point calibration range (0.4–200 μM) of PCE, TCE, trans-DCE, cis-DCE, and 1,1-DCE. PCBs were extracted from 1 mL cultures and analyzed using previously described methods (16, 19). All PCBs used in the cultures and as gas chromatography (GC) standards were of the highest available purity (≥99%) and were obtained from AccuStandard (New Haven, CT, USA).

### Enumeration of DF-1 and DSV by quantitative PCR (qPCR)

A 100 µL aliquot of the culture was diluted in 1 mL of Milli-Q water (Millipore) and centrifuged at 16,000 × *g* for 10 min at room temperature. The resulting cell pellet was resuspended in 100 µL of InstaGene Matrix (Bio-Rad Laboratories), and DNA was purified according to the manufacturer’s instructions.

DF-1 was enumerated by qPCR using the iQ SYBR Green Supermix (Bio-Rad Laboratories) and the 348F/884R primer set, as previously described (28), with slight modifications. Each 25 µL reaction consisted of 1× iQ SYBR Green Supermix, 400 nM of forward and reverse primers, and 1 µL of extracted DNA. A plasmid containing the 16S rRNA gene of DF-1 was used to generate a standard curve covering six orders of magnitude, ranging from 3.4 × 10³ to 3.4 × 10⁹ 16S rRNA gene copies per mL.

For DSV, qPCR was performed using the dsr2060F/dsr4R primer set targeting the dissimilatory sulfite reductase beta subunit (*dsrB*) gene, as previously described (46). Each 25 µL reaction contained 1× iQ SYBR Green Supermix, 400 nM of forward and reverse primers, and 1 µL of extracted DNA. A plasmid containing the *dsr*B gene of *Pseudodesulfovibrio* sp. was used to construct the standard curve, which spanned six orders of magnitude (3.4 × 10³ to 3.4 × 10⁹ *dsrB* gene copies per mL).

### Preparation, fractionation, and recovery of biosurfactants from *Pseudodesulfovibrio* sp.

*Pseudodesulfovibrio* sp. was cultured to a final concentration of approximately 10^8^ cells/mL in 50 mL of medium. The cell lysis extract was prepared as previously described (11, 16, 47). Briefly, the entire culture (cells with medium) was autoclaved at 121°C for 30 min to achieve thermal cell disruption and release of intracellular contents. After cooling to room temperature, the autoclaved material was passed through a sterile 0.4-µm filter under anaerobic conditions, and the resulting cell-free filtrate was collected. Titanium (III) nitrilotriacetate (TiNTA; 0.5 mM) was added as a reductant, and the filtrate was added to E-Cl medium at 1% (vol/vol) to support DF-1 growth.

An acid precipitate of the *Pseudodesulfovibrio* sp.-autoclaved extract was prepared according to Makkar and Cameotra (30, 31). Briefly, the autoclaved DSV culture was centrifuged at 3,000 × *g* for 20 min, and the resulting cell-free supernatant was acidified to pH 2.0 using 6 N HCl. The supernatant was then incubated at 4°C overnight. The resulting precipitate was separated by centrifuging at 3,000 × *g* for 20 min. The pellet was resuspended in E-Cl media, adjusted to pH 6.8, and used in subsequent experiments.

### Qualitative analysis of biosurfactants in *Pseudodesulfovibrio* sp. extracts

A qualitative oil-spreading assay was used to detect the presence of surface-active compounds, such as biosurfactants, in the DSV extracts. For this assay, 20 mL of distilled water was added to a Petri dish (90 mm × 15 mm), and 20 μL of a commercial motor oil (Shell Formula Shell SAE 10W–30, Shell Oil Products US, 1 U.S. quart) was added to the water surface. Subsequently, 10 μL of the DSV extract was gently layered onto the crude oil surface. The water surface was then observed for the formation of an oil-free, clear zone, indicating surfactant activity and the potential presence of biosurfactants. Controls were included to validate the assay, with sodium dodecyl sulfate (SDS) as the positive control, and distilled water as the negative control. The assay was performed in triplicate (48).

The emulsification index (% EI24) was determined to evaluate the emulsifying activity of the DSV extract. Equal volumes of crude oil and the DSV extract were mixed in a glass test tube (1:1 ratio) and vortexed for 2 min. The mixture was then left undisturbed at room temperature for 24 h. Following incubation, the height of the emulsion layer was measured. The emulsification index was calculated using the formula: % EI24 = (Height of the emulsion layer/Total height) × 100. Positive and negative controls, using SDS and distilled water, respectively, were included for validation (48). The assay was performed in triplicate.

### Identification and characterization of biosurfactants in DSV extract by HPLC-ESI-MS

Extracted lipids (0.5 mg) were resuspended at a concentration of 0.5 mg/mL in chloroform:methanol (1:1) with 200 µM butylated hydroxytoluene (BHT). Before analysis, the samples were further diluted 1,000-fold with a mixture of acetonitrile, isopropanol, and water (1:2:1, vol/vol/vol). Lipid extracts were analyzed by liquid chromatography coupled to high-resolution tandem mass spectrometry (LC-MS/MS). The LC-MS/MS analyses were performed on an Agilent 1290 Infinity LC coupled to an Agilent 6560 Quadrupole Time-of-Flight (Q-TOF) mass spectrometer. The separation was achieved using a C18 CSH (1.7 µm; 2.1 × 50 mm) column (Waters, Milford, MA). Mobile phase A was 10 mM ammonium formate with 0.1% formic acid in water/acetonitrile (40:60, vol/vol), and mobile phase B was 10 mM ammonium formate with 0.1% formic acid in acetonitrile/isopropanol (10:90, vol/vol). The gradient was held at 10% B for 1 min, ramped from 90% B over 5 min, held for 1 min, ramped to 10% B over 0.25 min, and held for 1.75 min to facilitate column equilibration. The flow rate was 0.4 mL/min. The column was maintained at 55°C, and the auto-sampler was maintained at 5 °C. A 2 µL injection was used for all samples. Mass spectrometry analysis was separated into two workflows: (i) high-resolution, accurate mass MS^1^ acquisition, and (ii) iterative MS/MS acquisition. The MS parameters for the iterative workflow were as follows: extended dynamic range, 2 GHz; gas temperature, 200°C; gas flow, 10 L/min; nebulizer, 345 kPa; sheath gas temperature, 300°C; sheath gas flow, 12 L/min; VCap, 3.0 kV (–); nozzle voltage, 250 V; *m/z* 119.0363, *m/z* 1033.9881 (–); MS and MS/MS Range, *m/z* 100–1700; acquisition rate, 3 spectra/s; isolation, narrow (~1.3 *m/z*); collision energy, 15 eV (–); max precursors/cycle, 3; precursor abundance-based scan speed, 25,000 counts/spectrum; ms/ms threshold, 5,000 counts and 0.001%; active exclusion enabled, yes; purity; stringency, 70%; cutoff, 0%; isotope model, and common organic molecules. The MS parameters for the MS^1^ workflow were the same for source and reference mass parameters and differed only for acquisition (selection of MS [same parameters], not Auto MS/MS). The LC-MS/MS data were processed using Agilent’s MassHunter Qualitative Browser (v 10.0).

### Quantification of rhamnolipids in *Pseudodesulfovibrio* sp. extracts

The anthrone-sulfuric acid assay was used to quantify rhamnolipid content in *Pseudodesulfovibrio* sp. extracts (49). A 0.2% (wt/vol) anthrone solution (Sigma-Aldrich) was prepared in concentrated sulfuric acid. Briefly, 1.25 mL of standard rhamnolipid solution or cell-free culture extract was added to 2.5 mL of anthrone-sulfuric acid reagent in a test tube on ice. The mixture was cooled in an ice-water bath for 10 min and then heated in a boiling water bath for 15 min. After heating, the test tube was cooled again in an ice-water bath. Upon development to a green-to-blue-green color, absorbance was measured at 625 nm using a spectrophotometer (Spectronic 21D, Milton Roy). The rhamnolipid concentration in the sample was determined using a standard curve prepared with commercially purified rhamnolipids from *P. aeruginosa* (>95% pure, AGAE Technologies) at concentrations ranging from 1 to 200 mg/L. The assay was performed in triplicate.

### Activity of DF-1 after substitution of DSV extracts with surfactants

Fifty milliliters of E-Cl medium supplemented with half-strength cysteine (1 mM) was anaerobically prepared in 160 mL serum bottles for DF-1 cultivation. In these experiments, the 1× *Pseudodesulfovibrio* sp. (DSV) extract was substituted with either extracted rhamnolipids, commercial rhamnolipids, or a commercial surfactin (> 98% pure, Sigma-Aldrich), at final concentrations ranging from 0.25 to 10 µg/mL. As electron acceptors, 2,3,4,5-tetrachlorobiphenyl (PCB 61, purity >99%, AccuStandard), 2,2′,3,3′,4,5,5′,6-octachlorobiphenyl (PCB 199, purity >99%, AccuStandard), or perchloroethene (PCE, purity >99%, Fluka) were added at final concentrations of 173 µM (PCB 61 and PCB 199) and 100 µM (PCE).

A total of 1 × 10⁵ DF-1 cells were inoculated into each 50 mL culture bottle. Negative controls received no rhamnolipids or DSV extract, while positive controls were supplemented with 1% (vol/vol) DSV extract. The pH of all rhamnolipid solutions was verified before inoculation to ensure consistency. Cultures were incubated at 29°C in the dark under static conditions. All experiments were performed in triplicate. Data were analyzed using nonlinear regression and plotted as a log (agonist) vs. response (three-parameter) curve in GraphPad Prism version 10. Dechlorination rates were calculated from the slope of a first-order linear regression, and the corresponding moles of chloride atoms removed in the presence of commercial rhamnolipid were also calculated.

### Bioinformatic and statistical analyses

The rhamnolipid synthesis pathway in *Pseudodesulfovibrio* sp. was investigated using the partially sequenced genome of *Pseudodesulfovibri*o sp. (unpublished data). Enzymes involved in rhamnolipid synthesis and their corresponding protein sequences were identified and extracted using SnapGene 8.0 (https://www.snapgene.com/). Sequence alignments were performed to analyze conservation across the bacterial kingdom using the UniProt database (https://www.uniprot.org/). The alignment results were visualized and annotated using Jalview (https://www.jalview.org/) to highlight conserved regions and sequence variations.

All statistical comparisons were performed relative to the negative control (no *Pseudodesulfovibrio* sp. extract or rhamnolipid supplementation) unless otherwise indicated, with full statistical details (test type, parameters, and *P* values) provided in the respective supplementary figure legends. Figures 3 to 5 were generated in Python using Matplotlib with SciPy (PCHIP interpolation), with data plotted as mean ± SD (*n* = 3), whereas Fig. S2 to S4 were prepared in GraphPad Prism (version 10).

## RESULTS

### Role of *Pseudodesulfovibrio* sp. extract in enhancing DF-1 growth-linked dechlorination of PCB 61: evidence of a lysis-released factor

As described by May et al. (11), DF-1 requires the amendment of the medium with an autoclaved, filter-sterilized extract from a *Pseudodesulfovibrio* sp. culture for growth-linked dechlorination of PCBs and PCEs after two sequential transfers in TiNTA-reduced medium. Based on these results, DSV extract (1% vol/vol) was used to culture DF-1 cells in the study, unless otherwise specified.

**Fig 1:**
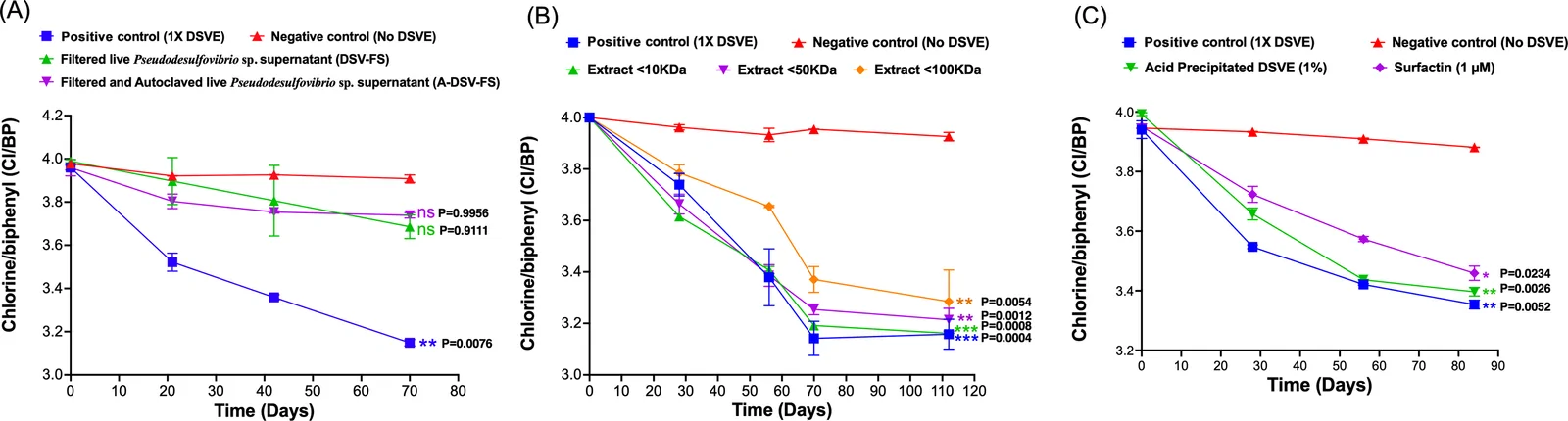
Characterization of the stimulatory factor in *Pseudodesulfovibrio* sp. extracts: non-secretory phenotype, molecular size, and biosurfactant-like properties. Panels show (**A**) comparison of PCB 61 dechlorination in DF-1 cultures supplemented with *Pseudodesulfovibrio* sp. extract (1× DSVE), filtered spent medium (DSV-FS), and autoclaved spent medium (A-DSV-FS); (**B**) filter-based molecular weight fractions of DSVE (<10 kDa, <50 kDa, and < 100 kDa) and their effect on DF-1 dechlorination activity; and (**C**) effect of acid-precipitated DSVE and a reference biosurfactant (surfactin, 1 µM) on DF-1 activity. In all experiments, 1× DSVE served as the positive control, and no DSVE served as the negative control. Abbreviations: DSVE, *Pseudodesulfovibrio* sp. extract; CI/BP, chlorine per biphenyl ratio. Data represent mean ± standard deviation from triplicate cultures. For each test condition, values were compared with the negative control (without 1× DSVE) using a two-tailed Welch’s *t*-test (unequal variances; difference defined as treatment, control; 95% CI; *n* = 3). Significance: *P < 0.05, **P < 0.01, ***P < 0.001.

To further elucidate the nature of the growth stimulant, DF-1 dechlorinating cultures were amended with one of three *Pseudodesulfovibrio* sp. preparations derived from cultures at ~10⁸ cells/mL: (i) filter-sterilized spent medium (DSV-FS), prepared by passing the culture through a 0.2-µm filter to remove intact cells, yielding a cell-free supernatant containing only secreted extracellular compounds; (ii) autoclaved filter-sterilized spent medium (A-DSV-FS), prepared identically to DSV-FS, but cell-free supernatant was autoclaved at 121°C for 30 min; or (iii) a cell lysis extract (DSV extract), prepared by autoclaving the entire culture (cells with media) at 121°C for 30 min to thermally lyse the cells and release intracellular contents, followed by 0.4-µm filtration to partially remove insoluble cell debris, with the resulting filtrate collected for use. After 20 days, cultures supplemented with DSV extract (positive control) showed a significant decline in the chlorine per biphenyl (CI/BP) ratio, dropping by approximately 20.5% (from 3.96 to 3.15), indicating robust dechlorination (*P* = 0.0076). In contrast, the DSV-FS and A-DSV-FS conditions exhibited only 7.6% and 6.4% reductions from their initial values, corresponding to less than 20–25% of the total dechlorination achieved with the DSV extract (Fig. 1A).

These findings demonstrate that the growth-promoting factor is retained within *Pseudodesulfovibrio* sp. cells and is released into the medium upon thermal lysis, as evidenced by the superior performance of the cell lysis extract (DSV extract) compared to the cell-free spent medium (DSV-FS) and autoclaved spent medium (A-DSV-FS), neither of which contains lysis-released intracellular contents. Notably, the negative control (no DSV extract) showed only minimal CI/BP reduction of PCB 61 (~1.8%), indicating that DF-1 alone lacks any appreciable intrinsic capacity to dechlorinate PCBs/PCEs in the absence of the lysis-derived *Pseudodesulfovibrio* sp. extract (Fig. 1A).

### Fractionation reveals a low-molecular-weight, thermostable factor in *Pseudodesulfovibrio* sp. extract

Filtration-based fractionation of the *Pseudodesulfovibrio* sp. extract (DSV extract) was performed using molecular weight cutoff filters of 10, 50, and 100 kDa to determine the size range in which the growth-enhancing molecule is retained (Fig. 1B). All three fractions enhanced DF-1 growth-linked dechlorination of PCB 61, but the magnitude varied. The <10 kDa fraction produced a ~14.8% reduction in the CI/BP ratio over 20 days (*P =* 0.0008), nearly matching the reduction observed with the unfractionated DSV extract (15.5%), indicating that the active compound is enriched in this range. The <50 kDa fraction retained measurable activity (15.4%) (*P =* 0.12), while the <100 kDa fraction showed reduced stimulation (8.7%) (*P =* 0.0054), suggesting possible dilution or masking by larger, inactive molecules. The fact that the strongest activity was consistently recovered in the <10 kDa fraction, closely paralleling the unfractionated control, demonstrates that the stimulatory molecule is 10 kDa or smaller.

Further biochemical characterization of the active <10 kDa fraction showed no reactivity with the Bradford reagent, confirming the absence of detectable proteinaceous material. Moreover, MALDI-TOF mass spectrometry did not identify any peptides within the active fraction (data not shown), reinforcing the conclusion that the stimulatory compound is non-proteinaceous.

The extract retained activity after autoclaving, indicating thermal stability, a feature characteristic of biosurfactants and other heat-resistant small metabolites. Given that biosurfactants enhance the solubilization and bioavailability of hydrophobic compounds, these observations are consistent with the active factor in the DSV extract being a low-molecular-weight biosurfactant that facilitates DF-1-mediated PCB and PCE dechlorination by improving substrate accessibility in aqueous systems.

### Acid-precipitated biosurfactant fraction facilitates DF-1 growth-linked dechlorination

To investigate the nature of the dechlorinating factor present in *Pseudodesulfovibrio* sp. extract (DSV extract), an acid precipitation extraction method was employed, as this technique selectively enriches for biosurfactants by lowering the pH to 2.0. Upon acidification with HCl, the visible precipitate was resuspended in E-Cl medium, which was then neutralized to pH 6.8. The acid-precipitated fraction facilitated significant dechlorination by DF-1 toward PCB congener PCB 61 over a 9- to 12-week period (Fig. 1C, *P =* 0.0026), albeit with slightly lower dechlorination than the unfractionated DSV extract.

To further assess whether the observed activity was due to a biosurfactant, surfactin, a well-characterized lipopeptide biosurfactant produced by *Bacillus subtilis,* was used as a reference compound. Treatment with 1 µM surfactin similarly stimulated dechlorination of PCB 61 by DF-1, supporting the hypothesis that the stimulatory effect in the DSV extract precipitate is attributable to biosurfactant activity (Fig. 1C).

**Fig 2:**
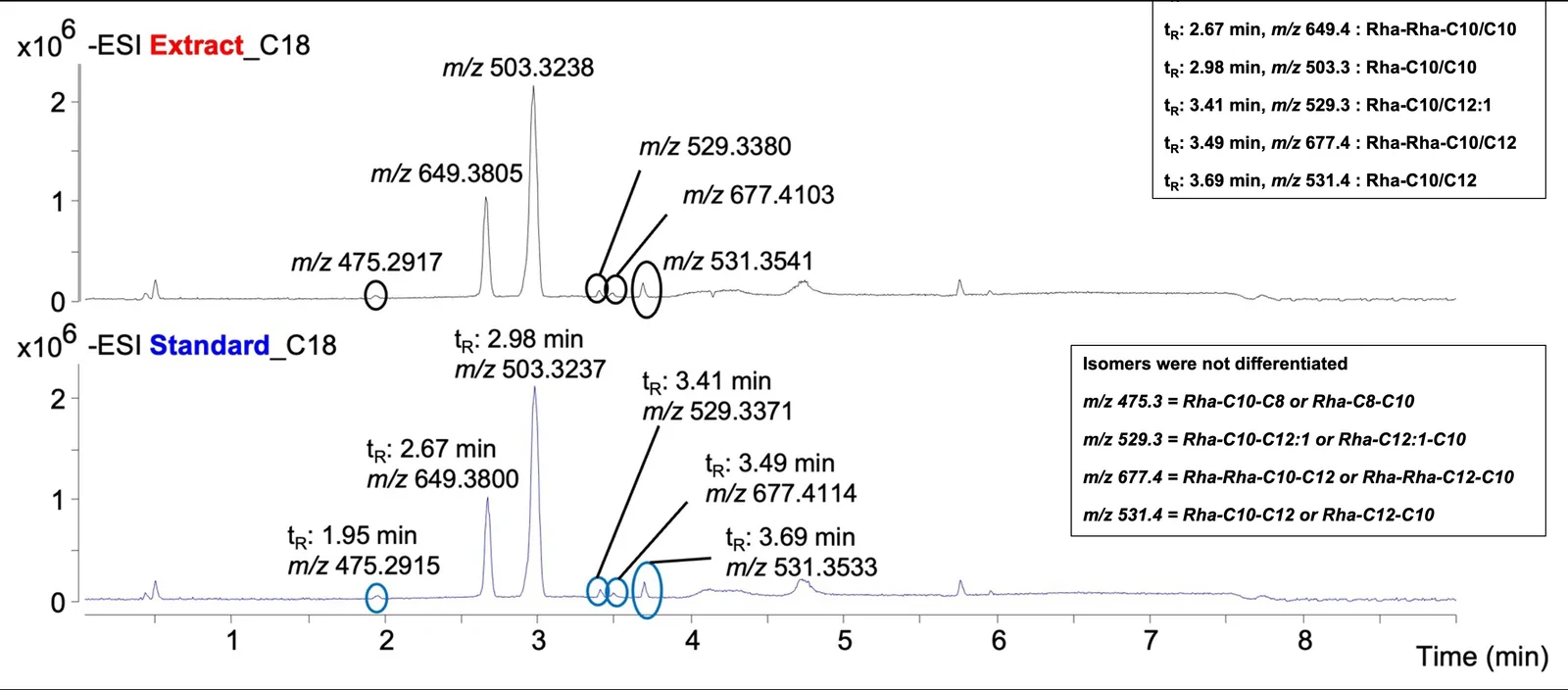
Identification of rhamnolipid congeners in *Pseudodesulfovibrio* sp. extract by LC-ESI-MS. Liquid chromatography-electrospray ionization-mass spectrometry analysis of the acid-precipitated *Pseudodesulfovibrio* sp. extract detected multiple pseudomolecular ions characteristic of mono- and di-rhamnolipids. Major ions were observed at *m/z* 475.3 (Rha-C10-C8/C8-C10; t_R_ 1.95 min), *m/z* 503.3 (Rha-C10-C10; t_R_ 2.98 min), *m/z* 529.3 (Rha-C10-C12:1/C12:1-C10; t_R_ 3.41 min), *m/z* 531.4 (Rha-C10-C12/C12-C10; t_R_ 3.69 min), *m/z* 649.4 (Rha-Rha-C10-C10; t_R_ 2.67 min), and *m/z* 677.4 (Rha-Rha-C10-C12/C12-C10; t_R_ 3.49 min). MS/MS fragmentation patterns supported structural assignments. Isomeric forms were not differentiated. Abbreviations: Rha, rhamnose; t_R_, retention time; LC-ESI-MS, *m/z*, mass/charge.

Genome analysis of *Pseudodesulfovibrio* sp. revealed the presence of a highly conserved rhamnolipid biosynthetic pathway, consistent with the production of glycolipid biosurfactants (Fig. S1A through D). To verify rhamnolipid production, the acid-precipitated fraction of the autoclaved *Pseudodesulfovibrio* sp. extract was analyzed by LC-ESI-MS and compared directly with a commercial rhamnolipid standard. The chromatographic profiles of the extract and the standard were highly similar, with overlapping retention times and comparable mass spectra. The analysis detected multiple precursor ions characteristic of rhamnolipids, including ions at *m/z* 475, 503, 529, 531, 649, and 677, corresponding to mono- and di-rhamnolipid congeners with C8–C12 acyl chains (Fig. 2). Structural assignments were confirmed by tandem MS fragmentation patterns consistent with previously reported rhamnolipid spectra (50). Isomeric forms were not distinguished. The close similarities between the extract and the standard are consistent with the observation that *Pseudodesulfovibrio* sp. produces a mixture of mono- and di-rhamnolipids. These glycolipid biosurfactants are known to enhance the solubilization and microbial accessibility of hydrophobic compounds, supporting their proposed role as major contributors to the growth-stimulating factor required by *D. chlorocoercia* DF-1.

Physicochemical analysis of the biosurfactant-enriched fraction further confirmed surfactant activity. The oil spreading diameter at 200 µg/mL of the extracted rhamnolipids was 1.1 ± 0.1 cm, which was not statistically different from that of a commercial rhamnolipid standard (1.4 ± 0.3 cm). Similarly, the extract’s emulsification index (%EI24) was 35.10%, compared to 43.33% for the standard, and this difference was also not statistically significant (*P* > 0.05). These results confirm that the precipitated material retains functional surface-active properties, although with slightly reduced efficacy relative to the commercial benchmark (Table S1).

Quantification of rhamnolipids using the anthrone-sulfuric acid assay revealed concentrations of approximately 20 µg/mL in the 100× concentrated extract and ~0.25 µg/mL in the 1× extract. These findings support the conclusion that rhamnolipid-based biosurfactants are enriched in the acid-precipitated fraction and play a pivotal role in enhancing the bioavailability of PCB 61 for microbial dechlorination by DF-1. Because these analyses are based on comparisons with available standards, it is not possible to exclude the presence of additional DSV cellular components that could contribute to enhanced DF1 growth.

### Biosurfactants stimulate PCE growth-linked dechlorination-l of *D. chlorocoercia*

The effect of extracted rhamnolipids and commercial rhamnolipids on the growth-linked dechlorination activity of DF-1 was evaluated across a concentration range of 0.25–10 µg/mL using PCE as the electron acceptor at a concentration of 100 µM. Dechlorination performance was measured by quantifying the transformation products of PCE, trichloroethene (TCE), cis-1,2-dichloroethene (cDCE), and trans-1,2-dichloroethene (tDCE) via gas chromatography. The concentration range of rhamnolipids tested was based on the measured quantity of rhamnolipids in 1× and 5× DSV extract.

**Fig 3:**
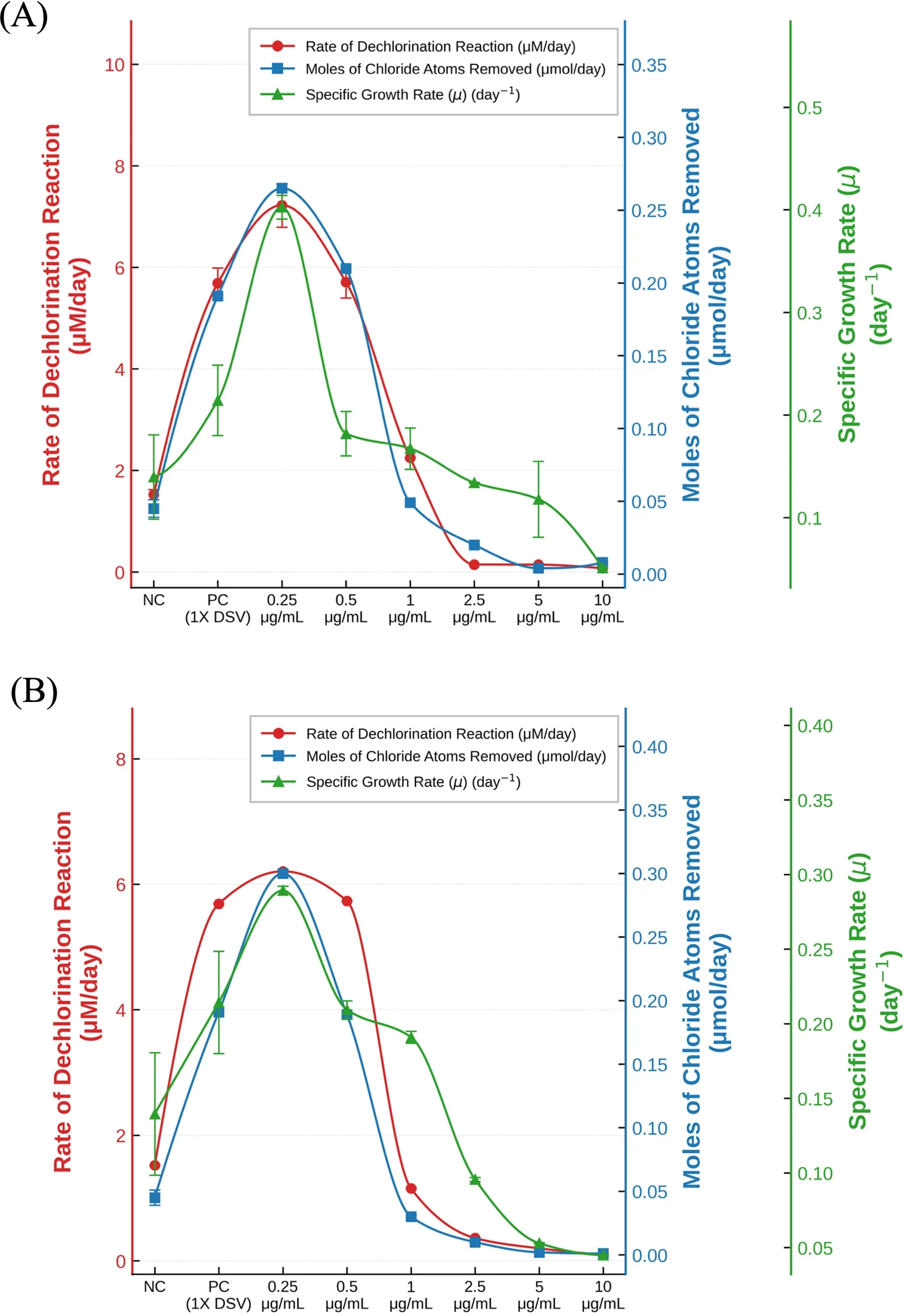
Effect of rhamnolipid concentration on PCE dechlorination and growth of *D. chlorocoercia* DF 1. Concentration-dependent effects of (**A**) commercial rhamnolipids and (**B**) extracted/enriched rhamnolipids (0.25–10 µg/mL) on DF 1 activity are shown using 100 µM PCE as the electron acceptor. Cultures were compared to positive controls containing 1% (vol/vol) *Pseudodesulfovibrio* sp. extract and negative controls lacking rhamnolipid or extract supplementation. Line graphs depict the rate of dechlorination, the moles of chlorine atoms removed, and the specific growth rate across rhamnolipid concentrations, illustrating a concentration-dependent response with maximal stimulation at submicellar concentrations (0.25–0.5 µg/mL) and inhibition at higher concentrations. Data represent mean values from triplicate cultures.

DF-1 cultures supplemented with extracted rhamnolipids or commercial rhamnolipids displayed a concentration-dependent response in dechlorination activity. Low rhamnolipid concentrations (0.25–0.5 µg/mL) enhanced PCE dechlorination rates beyond those observed in the positive control (DSV extract), with an average of 4%–5% increase. At 1.0 µg/mL, dechlorination activity remained strong but declined slightly, showing a 9%–12% reduction compared to the positive control. A marked decrease in activity was observed at concentrations above 1.0 µg/mL, indicating a threshold beyond which rhamnolipids exert an inhibitory effect (Fig. 3A and B; Fig. S2A and B).

Nonlinear regression analysis revealed a sigmoidal dose-response curve, indicating that the most effective rhamnolipid concentrations lie within the 0.25–0.5 µg/mL range. At these levels, DF-1 cultures exhibited the highest dechlorination rates and the most chlorine atoms removed per mole of PCE, supporting the role of biosurfactants in enhancing electron acceptor availability (Fig. 3A and B; Fig. S2A and B).

Enumeration of DF-1 cells revealed that growth closely mirrored PCE dechlorination across rhamnolipid concentrations, with peak cell densities at 0.25–1.0 µg/mL and cultures lacking rhamnolipids exhibiting minimal dechlorination and cell numbers near the qPCR detection limit. This strong correlation between dechlorination and biomass, together with the constant TiNTA concentration in all treatments, indicates that enhanced dechlorination is primarily due to rhamnolipid-mediated improvement of PCE bioavailability and reflects DF-1-mediated reductive dechlorination rather than treatment-specific abiotic reactions between PCE and Ti(III) (Fig. 3A and B; Fig. S2C and D).

### Rhamnolipids stimulate the growth-linked dechlorination of PCB 61 and PCB 199 by DF-1 in a concentration-dependent manner

**Fig 4:**
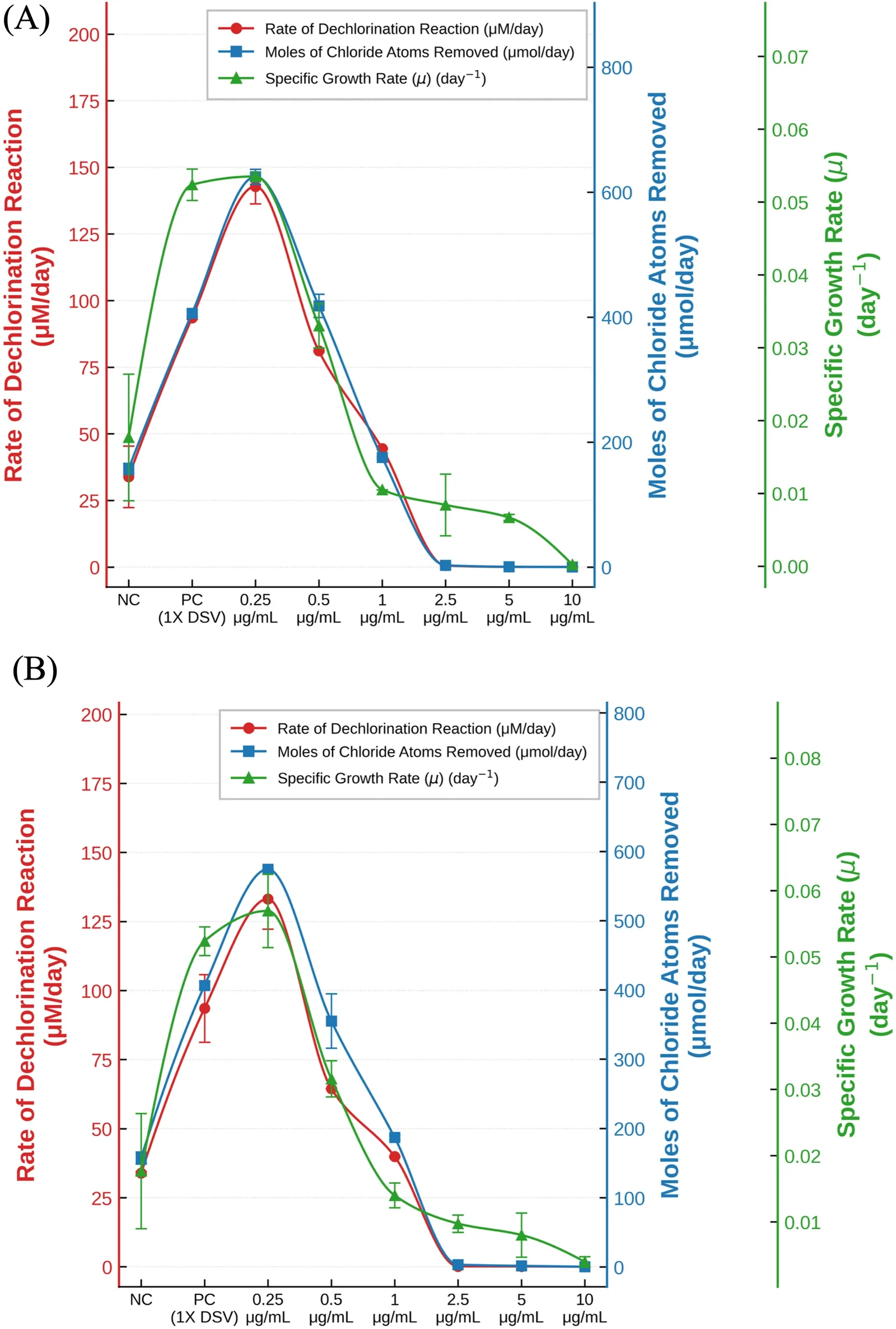
Effect of rhamnolipid concentration on PCB 61 dechlorination and growth of *D. chlorocoercia* DF-1. Concentration-dependent effects of (**A**) commercial rhamnolipids and (**B**) extracted/enriched rhamnolipids (0.25–10 **µ**g/mL) on DF-1 activity are shown using PCB 61 as the electron acceptor and PCB 23 as the principal dechlorination product. Line graphs depict dechlorination rate, moles of chlorine atoms removed, and specific growth rate across rhamnolipid concentrations relative to positive controls containing 1% (vol/vol) *Pseudodesulfovibrio* sp. extract and negative controls lacking rhamnolipid or extract supplementation. Rhamnolipid addition enhanced DF-1 dechlorination and growth in a concentration-dependent manner, with maximal stimulation at low (0.25–0.5 µg/mL) concentrations and diminished activity at higher doses, indicating inhibitory effects. Data represent mean values from triplicate cultures.

The growth-linked dechlorination activity of DF-1 varied markedly with rhamnolipid concentrations ranging from 0.25 to 10 µg/mL. PCB 61 was supplied as the electron acceptor, with PCB 23 as the principal dechlorination product. At the lowest concentration tested (0.25 µg/mL), DF-1 exhibited the highest total dechlorination, with PCB 23 accumulating at a rate exceeding 140 nM/day and chloride release exceeding 600 µmol Cl^−^/day in 50 mL cultures. This represents a significantly greater activity than the positive control (*P =* 0.0008), which showed approximately 90 nM/day PCB 23 accumulation (a ~35% reduction) and approximately 406 µmol Cl^−^ released/day (a ~35% reduction). A moderate response was observed at 0.5 µg/mL, where the dechlorination rate declined to ~80 nM/day (a 44% reduction relative to 0.25 µg/mL) with 418 µmol Cl^−^ released/day (a 33% reduction), suggesting partial inhibition or suboptimal stimulation (Fig. 4; Fig. S3A and B).

At 1 µg/mL, dechlorination activity was strongly suppressed, with PCB 23 accumulation decreasing to ~40 nM/day (a ~70% decline) and chloride release reduced by ~71%. At higher rhamnolipid concentrations (2.5–10 µg/mL), dechlorination was minimal, with PCB 23 levels remaining <10 nM and rates <1 nM/day. These conditions also resulted in markedly lower cell numbers (Fig. 4; Fig. S3C and D). Collectively, these findings indicate that a rhamnolipid concentration of 0.25–0.5 µg/mL is the optimal range for enhancing DF-1 activity, thereby effectively promoting PCB 61 bioavailability and dechlorination without adverse effects. Conversely, excess rhamnolipids inhibit dechlorination, underscoring the importance of precise dosage for maximizing biomass yields in DF-1 (Fig. 4).

**Fig 5:**
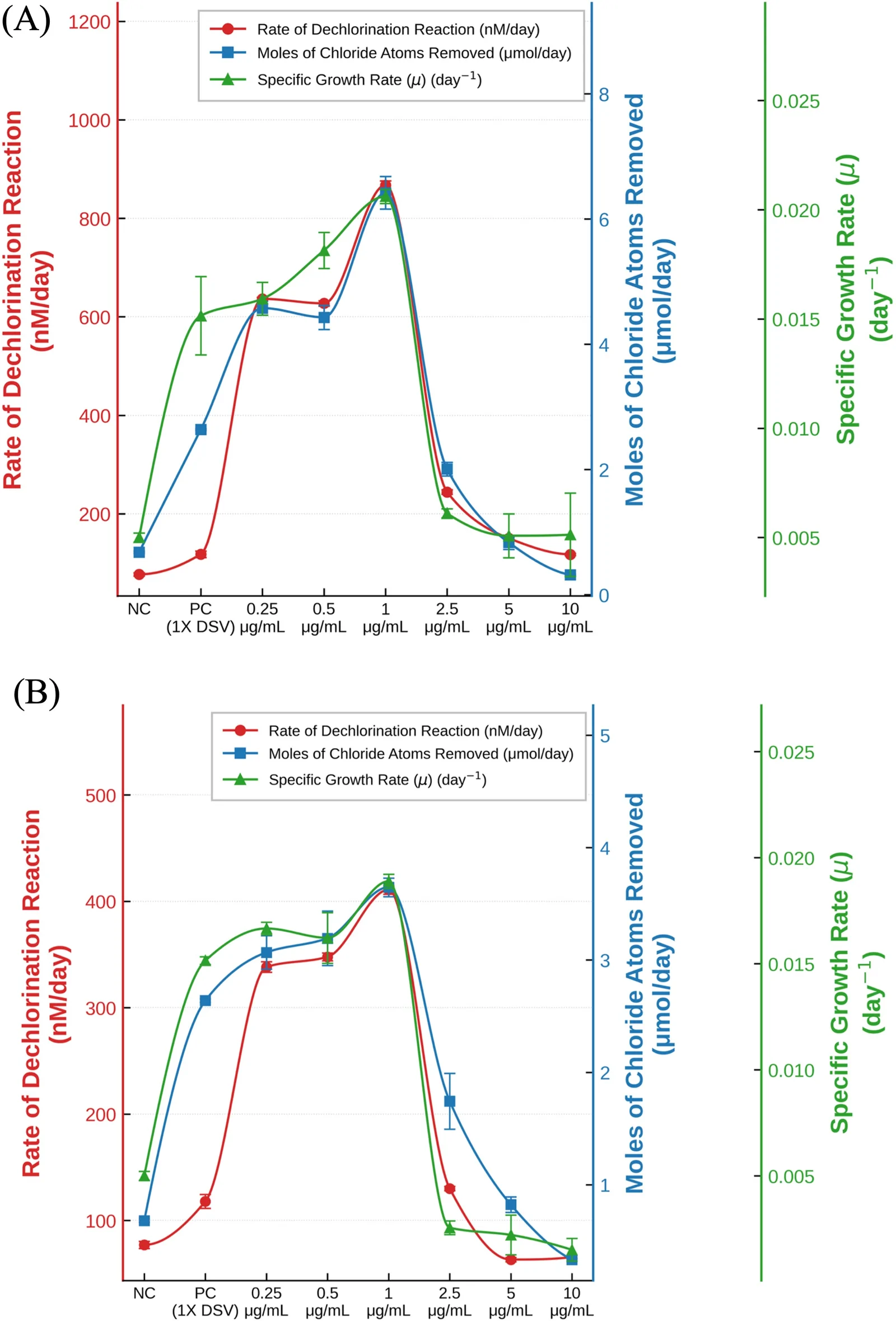
Effect of rhamnolipid concentration on PCB 199 dechlorination and growth of *D. chlorocoercia* DF-1. Concentration-dependent effects of (**A**) commercial rhamnolipids and (**B**) extracted/enriched rhamnolipids (0.25–10 µg/mL) on DF-1 activity are shown using PCB 199 as the electron acceptor and PCB 178 as the principal dechlorination product. Line graphs depict dechlorination rate, moles of chlorine atoms removed, and specific growth rate across rhamnolipid concentrations relative to positive controls containing 1% (vol/vol) *Pseudodesulfovibrio* sp. extract and negative controls lacking rhamnolipid or extract supplementation. Rhamnolipid addition enhanced DF-1 dechlorination and growth in a concentration-dependent manner, with maximal stimulation at 1.0 µg/mL and diminished activity at higher doses, indicating inhibitory effects at supra-optimal concentrations. Data represent mean values from triplicate cultures.

Interestingly, the optimal rhamnolipid concentration shifted significantly when DF-1 was challenged with a more extensively chlorinated congener, PCB 199, which yields PCB 178 as the dechlorination product (Fig. 5A and B; Fig. S4A and B). Although lesser chlorinated congeners such as PCE and PCB 61 exhibited maximum dechlorination at 0.25 µg/mL, PCB 199 dechlorination was maximized at 1 µg/mL of rhamnolipids. At this concentration, DF-1 achieved the highest dechlorination rate (~1.15 nM/day) with a chloride release of approximately 3.2 µmol/day. In comparison, 0.25 and 0.5 µg/mL rhamnolipid yielded a lower rate of dechlorination, with reductions of 26% (0.85 nM/day; 2.29 µmol Cl^−^/day) and 51% (0.57 nM/day; 1.87 µmol Cl^−^/day), respectively. Despite these reductions, DF-1 cultures treated with 0.25–1 µg/mL rhamnolipids exhibited substantially higher activity than the positive control, highlighting the stimulatory effect of rhamnolipids on DF-1-mediated dechlorination of more extensively chlorinated congeners (Fig. 5A and B). However, as observed with PCB 61, rhamnolipid concentrations above the optimum (>1 µg/mL) resulted in a sharp decline in both dechlorination rates and cell biomass yields, indicating inhibitory effects beyond the stimulatory window (Fig. 5A and B; Fig. S4A through D). Notably, although rhamnolipids significantly enhanced DF-1 activity for both PCB 61 and PCB 199, the overall dechlorination rate for the more extensively chlorinated congener (PCB 199; ~1.15 nM/day) remained markedly lower than that observed for PCB 61 (~140 nM/day). This underscores the inherent kinetic limitations of congener structure, with more extensively chlorinated PCBs exhibiting slower kinetics despite biosurfactant facilitation. Rhamnolipid consistently outperformed the *Pseudodesulfovibrio* sp. cell-free extract at comparable concentrations (Fig. 3 to 5). If other cellular components, such as phospholipids, contributed substantially to stimulation, the crude extract would be expected to produce a greater effect than rhamnolipid alone; however, this was not observed, indicating that the stimulatory activity is not due to a general surfactant effect of cellular debris but is largely associated with rhamnolipid-type glycolipid biosurfactants. Nevertheless, contributions from other co-extracted glycolipids cannot be excluded. In contrast, the negative control receives only the inoculum DF-1 cells and must rely on slow solubilization of the hydrophobic PCB electron acceptors from the undissolved reservoir into the aqueous phase; hence, DF-1 can grow under these conditions, but growth in the absence of rhamnolipids or extract is slow and limited, as reflected by the markedly lower maximum cell numbers compared with rhamnolipid-amended treatments (Fig. 4 and 5; Fig. S4C and D). Rhamnolipids, therefore, do not create *de novo* growth; instead, they shorten the lag and substantially increase the rate and extent of DF-1 biomass accumulation by enhancing PCB bioavailability.

## DISCUSSION

Reductive dechlorination of PCBs and PCEs under anaerobic conditions has been extensively studied in sediment-enriched microbial consortia (4, 10, 51); however, investigations using defined cultures remain limited. Among the few well-characterized isolates, *Dehalococcoides* spp. including *D. mccartyi* strains 195 (52), CBDB1 (53), BAV1 (54), GT (54), and FL2 (55) are capable of dechlorinating PCE, TCE, DCE, vinyl chloride, and, in some cases, PCBs, with CBDB1 being the best-characterized for PCB dechlorination. Additionally, the *D. chlorocoercia* strain DF-1, isolated from Charleston Harbor sediment, has demonstrated activity against PCBs and PCE (11, 17, 24). Notably, DF-1 requires supplementation with autoclaved, filtered cell extract from *Pseudodesulfovibrio* sp. to dechlorinate PCB congeners and PCE, indicating the presence of a critical stimulatory factor (11, 17). Only extracts derived from sulfate-reducing bacteria (SRB) are reported to elicit this stimulatory effect on DF-1 (11). Previous studies and current findings indicate that SRBs neither utilize PCBs or PCEs as terminal electron acceptors nor do they cometabolize them in any form; nevertheless, their presence or the presence of cellular lysate appears essential for efficient DF-1-mediated growth-linked dechlorination (11, 16, 17, 24).

In both natural and enriched systems, *Dehalococcoides* spp. commonly coexist with fermentative and syntrophic bacteria, such as *Desulfovibrio*, *Eubacterium*, *Acetobacterium*, *Citrobacter*, and *Clostridium*, which provide hydrogen, acetate, and corrinoids essential for organohalide respiration (56–58). While *Pseudodesulfovibrio* sp. can grow independently, DF-1 depends on its cell lysate for growth-linked dechlorination, indicating a unidirectional syntrophic interaction shaped by shared anoxic niches and long-term coevolution. Notably, even supplementation with electron donors, cofactors, and coenzymes fails to substitute this dependency, suggesting that the stimulatory factor provided by *Pseudodesulfovibrio* sp. is not a conventional nutritional requirement for DF-1 but instead plays a vital role in enhancing dechlorination activity. Current findings reinforce this commensal dependency, revealing that the thermostable stimulatory factor (<10 kDa) is not actively secreted but becomes bioavailable upon cell lysis, likely explaining why autoclaved or lysed *Pseudodesulfovibrio* sp. preparations consistently stimulate DF-1 activity (11). One plausible explanation is that the faster-growing *Pseudodesulfovibrio sp.* cells reach the stationary or death phases earlier than DF-1, releasing intracellular compounds required for DF-1 function. Lysis may occur due to nutrient limitation, microbial competition, predation (e.g., by protozoa or bacteriophages), or redox stress. Alternatively, the active compound may be membrane-associated and released only upon structural disruption.

Physicochemical characterization indicated that the stimulatory factor is lipid-based. To verify that the observed surface activity was attributable to biosurfactant content rather than to nonspecific amphiphilic bacterial components, the active <10 kDa fraction was assessed for proteinaceous material before further characterization. Its small size (<10 kDa), thermostability, lack of reactivity with the Bradford reagent, and absence of peptide signals by MALDI-TOF mass spectrometry (data not shown) collectively excluded a proteinaceous identity, ruling out foaming proteins or amphiphilic peptides released during cell lysis as contributors to the observed surface activity. Distilled water served as the negative control for both the oil-spreading and emulsification assays, establishing the baseline against which surfactant activity was assessed. Consistent with this, acidification of the *Pseudodesulfovibrio* sp. extract to pH 2 yielded a precipitate that, upon neutralization, restored growth-linked dechlorination activity and displayed oil-spreading and emulsification, hallmarks of biosurfactants. A parallel treatment with 1 µM surfactin, a lipopeptide biosurfactant from *Bacillus subtilis*, produced a similar stimulatory effect on DF-1, reinforcing the biosurfactant hypothesis. LC-MS analysis detected ions and fragmentation patterns in the acid-precipitated fraction that were consistent with rhamnolipid congeners, and genome analysis revealed a conserved rhamnolipid biosynthetic pathway in *Pseudodesulfovibrio* sp. (Fig. S1A through D). The 1× extract contained approximately 0.25 µg/mL rhamnolipid-equivalent glycolipids based on quantification by the anthrone-sulfuric acid assay. These findings align with reports that rhamnolipids and other biosurfactants are commonly retained within membranes or intracellular inclusions under anaerobic conditions and are released primarily upon cell disruption (30–32). Autoclaving facilitates this release by rupturing cells, and the persistence of activity after heat treatment highlights the thermostable nature of rhamnolipids (31, 37, 38). Acid precipitation further enriched the factor by promoting glycolipid aggregation at low pH, a well-established recovery method (59, 60). Collectively, these physicochemical properties and functional assays are consistent with a DF-1 stimulatory factor that is dominated by rhamnolipid-type glycolipid biosurfactants.

Biosurfactants are widely recognized for enhancing the microbial degradation of hydrophobic pollutants, such as PCBs, by improving substrate bioavailability. Their amphiphilic nature facilitates pseudosolubilization, reducing the size of hydrophobic compound droplets to < 1 µm, enhancing microbial uptake. Multiple studies have shown that surfactants enhance the biodegradation of recalcitrant compounds through emulsification, micellar solubilization, desorption of bound pollutants, and modulation of microbial adhesion (35, 36, 40, 41, 61, 62).

Given the low solubility of PCBs, their microbial utilization is often limited by inefficient substrate transfer. Biosurfactant-producing microbes overcome this barrier through two main strategies: direct contact with pseudosolubilized droplets and biosurfactant-mediated transport (30, 61). These findings support the conclusion that the DF-1 stimulatory factor in *Pseudodesulfovibrio* sp. extract is partially or wholly mediated by rhamnolipid-type glycolipid biosurfactants that enhance PCB bioavailability, thereby facilitating DF-1-mediated growth-linked reductive dechlorination. Importantly, the stimulatory effect observed in this study cannot be attributed to a nonspecific surfactant contribution from cellular components, such as phospholipids, present in the *Pseudodesulfovibrio* sp. extract. If such components played a dominant role, cell-free extracts would be expected to produce greater enhancement of DF-1 growth-linked dechlorination than purified rhamnolipids. However, rhamnolipids alone consistently produced stronger stimulation than the extract at comparable concentrations (Fig. 3 to 5), suggesting that the observed effect is attributable solely to rhamnolipids.

For an obligate organohalide-respiring bacterium such as *D. chlorocoercia* DF-1, energy conservation is tightly coupled to the flux of electrons from formate to halogenated electron acceptors via reductive dehalogenation. Rhamnolipids increase the effective concentration of PCE and PCBs at the cell surface through pseudosolubilization and microphase formation, thereby reducing substrate limitation and enabling DF-1 to sustain higher respiratory rates and biomass at a given formate supply. Although substrate uptake is likely passive, this enhanced electron-acceptor availability effectively increases the rate and extent of energy conservation per unit time.

To investigate the dose-dependent effects of rhamnolipids on DF-1 growth-linked dechlorination, both commercial and *Pseudodesulfovibrio* sp.-derived rhamnolipids were tested across a concentration range using PCE, PCB 61, and PCB 199 as electron acceptors. These compounds vary in hydrophobicity, with PCE (*logK_ow_* = 2.88) being relatively hydrophilic, PCB DF61 (*logK_ow_* = 6.04) moderately hydrophobic, and PCB 199 (*logK_ow_* = 7.20) highly hydrophobic, which influenced the concentration of rhamnolipid required for effective dechlorination (3). At 0.25 µg/mL, rhamnolipids supported maximal DF-1 growth and dechlorination of PCE and PCB 61, while PCB 199 required a higher concentration (1 µg/mL) to achieve similar activity. This shift in optimal concentration parallels the increasing hydrophobicity of the electron acceptors. It is consistent with rhamnolipids acting as solubilizing agents that enhance the bioavailability of otherwise poorly soluble substrates in a dose-dependent manner.

Interestingly, this effect was biphasic: optimal concentrations improved growth-linked dechlorination, whereas higher concentrations exerted pronounced inhibitory effects. This suggests a complex interplay between rhamnolipid concentration, substrate accessibility, and microbial physiology. At submicellar levels (e.g., 0.25–1 µg/mL), rhamnolipids likely facilitate substrate uptake by reducing surface tension and pseudosolubilizing hydrophobic molecules, enhancing DF-1’s access to electron acceptors. In addition to solubilization, rhamnolipids have also been reported to influence redox processes and biofilm structure in other systems. Pham et al. (63) demonstrated that rhamnolipids, in conjunction with phenazine-1-carboxamide, enhanced extracellular electron transfer in microbial fuel cells (63). Similarly, rhamnolipids have also been shown to modulate iron-based abiotic dechlorination systems: Basnet et al. (64) demonstrated that they effectively stabilize Pd-doped nZVI, enhancing transport in porous media (64), while Bhattacharjee et al. (65) reported that rhamnolipid-coated Pd-nZVI altered trichloroethene dechlorination kinetics in a biphasic manner, collectively suggesting a role in mediating interfacial and redox processes (65, 66).

Although PCE exhibits a much higher aqueous solubility (~0.15 g/L) than PCB 61 (0.16 × 10⁻⁴ g/L) (67, 68), rhamnolipids still enhanced growth-linked dechlorination. PCB has a higher partitioning coefficient (K_ow_ = 6.04) than PCE (K_ow_ = 2.88), but PCE remains hydrophobic, with a maximum aqueous solubility of only ~0.9 mM. In anaerobic batch cultures, PCE’s high volatility and elevated Henry’s law constant promote rapid partitioning into the headspace, progressively decreasing its availability to DF-1 over extended incubations (69–71). Under these conditions, rhamnolipids function as solubilizing biosurfactants that increase the effective bioavailability of PCE by emulsification and microphase formation, thereby reducing substrate limitation at high cell densities. This enhanced electron acceptor availability leads to increased PCE dechlorination rates and higher DF-1 biomass in a concentration-dependent manner (72). Abiotic reduction of chloroethenes by reduced metals is known to be chemically feasible (73, 74), and Ti (III) was used as the reductant in these experiments (75). In the present work, TiNTA was maintained at a constant concentration across all treatments; hence, any abiotic reaction between PCE and Ti (III) would occur equally in all bottles. The pronounced, sigmoidal increase in both dechlorination rates and DF-1 cell biomass with increasing rhamnolipid concentration, relative to the low-activity controls, therefore reflects a biological response to enhanced PCE bioavailability rather than differences in abiotic PCE loss.

Rhamnolipids are also known to solubilize hydrophobic compounds below their critical micelle concentration (CMC), which ranges from 53 µg/mL to 230 µg/mL (76–78). In this study, the concentrations that stimulated dechlorination were significantly lower than the CMC values, further supporting a mechanism driven by submicellar interactions. This aligns with the hypothesis that rhamnolipids released upon *Pseudodesulfovibrio* sp. cell lysis enhance substrate transfer to DF-1 even at low concentrations.

However, at supra-optimal concentrations, two non-exclusive inhibitory mechanisms may arise. The first is cytotoxicity: elevated rhamnolipid levels can disrupt membrane integrity or induce envelope stress, impairing essential cellular functions such as transport, respiration, and energy generation. Rhamnolipids, being amphiphilic molecules, integrate into the lipid bilayer and perturb membrane architecture, increasing permeability and potentially leading to ion leakage and loss of proton motive force. This membrane disruption can trigger envelope stress responses and modulate redox-sensitive regulatory systems (77–82). Second, substrate sequestration: rhamnolipids may encapsulate or micellarize hydrophobic electron acceptors at high concentrations, sequestering them away from the DF-1 cell surface and reducing their adequate bioavailability (72, 83, 84).

These findings highlight that while rhamnolipid-type biosurfactants significantly enhance DF-1 growth-linked dechlorination at optimal concentrations, careful calibration is essential. Excessive doses not only reduce electron acceptor bioavailability through micellar entrapment but may also compromise microbial viability and respiratory function. Thus, the dual role of rhamnolipids, as both facilitators and inhibitors, must be considered when leveraging them for the growth of organohalide-respiring bacteria like DF-1.

### Future objectives

Rhamnolipids hold significant potential to improve the scale-up of DF-1 at high volumes by enhancing growth, substrate solubility, and culture homogeneity, which are essential for producing high-density, reproducible inocula for field applications. Additionally, the amphiphilic nature of rhamnolipids may improve DF-1 adherence to activated carbon, a technology already in use for field deployment at PCB-contaminated sites. This combination of DF-1, rhamnolipids, and activated carbon could form a synergistic platform that enhances PCB bioavailability and accelerates site-specific dechlorination. Future studies should optimize rhamnolipid concentrations to maximize bacterial colonization and activity while ensuring environmental compatibility.

## Data Availability

Data will be made available on request.

## SUPPLEMENTAL MATERIAL

Supplemental material aem.00841-26-s0001.docx Fig. S1 to S4; Table S1.

ASM does not own the copyrights to Supplemental Material that may be linked to, or accessed through, an article. The authors have granted ASM a non-exclusive, world-wide license to publish the Supplemental Material files. Please contact the corresponding author directly for reuse.
