# Supplementary material for: Rhamnolipid-augmented medium enhances growth of Dehalobium chlorocoercia on chloroethenes and polychlorinated biphenyls
Source: Appl Environ Microbiol. 2026 Aug 3;92(8):e00841-26. doi: 10.1128/aem.00841-26 (PMC13488350; doi:10.1128/aem.00841-26)
Supplement: Supplementary file 1 [file aem.00841-26-s0001.docx]

**Supplementary Materials**

**Rhamnolipid-Augmented Medium Enhances Growth of Dehalobium chlorocoercia on Chloroethenes and Polychlorinated Biphenyls**

Vijay Hemmadi ^a^, Randhir S. Makkar ^b^, Jace W. Jones ^c^, Christopher W. Marshall ^d^, Allen R. Place ^e^, Harold D. May ^f^, and Kevin R Sowers ^a,#^

^a^ Department of Marine Biotechnology, Institute of Marine and Environmental Technology, University of Maryland Baltimore County, Baltimore, MD, USA

^b^ Enzylignol Inc, 279 Sweet Alyssum Drive, Ladson, SC, USA

^c^ Department of Pharmaceutical Sciences, University of Maryland School of Pharmacy, Baltimore, MD, USA

^d^ Department of Biological Sciences, Marquette University, Milwaukee, WI, USA

^e^ University of Maryland Center for Environmental Sciences, Institute of Marine and Environmental Technology, Baltimore, MD, USA

^f^ Hollings Marine Laboratory, Medical University of South Carolina, Charleston, SC, USA

Running Head: Rhamnolipids promote *Dehalobium chlorocoercia* growth and activity

#Address correspondence to Kevin R Sowers, Sowers@umbc.edu.

Number of figures: 4

Fig. S1A

Fig. S1B

Fig. S1C

Fig. S1D

**Fig. S1.** Identification of putative rhamnose biosynthesis genes in the Pseudodesulfovibrio sp. genome. Homologs of the canonical *rml* operon were detected by BLASTp using Pseudomonas aeruginosa sequences as queries. The identified genes encode: (A) RmlA, glucose-1-phosphate thymidylyltransferase (882 bp; 293 aa; 32.6 kDa); (B) RmlB, dTDP-D-glucose 4,6-dehydratase (338 aa; 37.9 kDa); (C) RmlC, dTDP-4-dehydrorhamnose 3,5-epimerase (189 aa; 21.2 kDa); and (D) RmlD, dTDP-4-dehydrorhamnose reductase (291 aa; 31.3 kDa). Conserved functional domains were verified by multiple sequence alignment, and phylogenetic analysis grouped the Pseudodesulfovibrio sp. homologs with known *rml* gene products. Together, these results indicate the presence of a complete dTDP-L-rhamnose biosynthetic pathway, supporting the potential for rhamnolipid production in Pseudodesulfovibrio sp.

**Fig. S2. Effect of rhamnolipid supplementation on PCE dechlorination and growth of DF-1.**
Panels show (A) commercial rhamnolipid (CRL) and (B) extracted/enriched rhamnolipid (ERL) treatments across 0.25–10 µg/mL; (C–D) DF-1 cell numbers determined by 16S rRNA gene copy number corresponding to CRL (C) and ERL (D). For each test condition, values were compared with the negative control (without 1X DSVE) using a two-tailed Welch’s t-test (unequal variances; difference defined as treatment, control; 95% CI; n = 3). Significance: *P < 0.05, **P < 0.01, ***P < 0.001.

**Fig. S3. Effect of rhamnolipid supplementation on PCB-61 dechlorination and growth of DF-1.**
Panels show (A) commercial rhamnolipid (CRL) and (B) extracted/enriched rhamnolipid (ERL) treatments across 0.25–10 µg/mL with PCB-23 as the principal dechlorination product; (C–D) DF-1 cell biomass corresponding to CRL (C) and ERL (D). For each test condition, values were compared with the negative control (without 1X DSVE) using a two-tailed Welch’s t-test (unequal variances; difference defined as treatment, control; 95% CI; n = 3). Significance: *P < 0.05, **P < 0.01, ***P < 0.001.

**Fig. S4. Effect of rhamnolipid supplementation on PCB-199 dechlorination and growth of DF-1.**
Panels show (A) commercial rhamnolipid (CRL) and (B) extracted/enriched rhamnolipid (ERL) treatments across 0.25–10 µg/mL with PCB-177 as the principal dechlorination product; (C–D) DF-1 cell biomass corresponding to CRL (C) and ERL (D). For each test condition, values were compared with the negative control (without 1X DSVE) using a two-tailed Welch’s t-test (unequal variances; difference defined as treatment, control; 95% CI; n = 3). Significance: *P < 0.05, **P < 0.01, ***P < 0.001.

**Table 1S. Surface-active properties of extracted and commercial rhamnolipids. Oil spreading diameter and emulsification index (EI24) were measured at 200 µg/mL. Values represent mean ± standard deviation from triplicate assays.**

| Sample | Oil spreading (cm) | Emulsification Index (%) |
| --- | --- | --- |
| 200 μg/mL commercial rhamnolipids | 1.4±0.3 | 43.33 |
| 200 μg/mL extracted rhamnolipids | 1.1±0.1 | 35.10 |
